# How do voice acoustics affect the perceived trustworthiness of a speaker? A systematic review

**DOI:** 10.3389/fpsyg.2025.1495456

**Published:** 2025-03-10

**Authors:** Constantina Maltezou-Papastylianou, Reinhold Scherer, Silke Paulmann

**Affiliations:** ^1^Department of Psychology and Centre for Brain Science, University of Essex, Colchester, United Kingdom; ^2^Brain-Computer Interfaces and Neural Engineering Laboratory, School of Computer Science and Electronic Engineering, University of Essex, Colchester, United Kingdom

**Keywords:** trust, speech acoustics, trustworthy voice, human-robot interaction, voice assistants, intelligent agents

## Abstract

Trust is a multidimensional and dynamic social and cognitive construct, considered the glue of society. Gauging someone’s perceived trustworthiness is essential for forming and maintaining healthy relationships across various domains. Humans have become adept at inferring such traits from speech for survival and sustainability. This skill has extended to the technological space, giving rise to humanlike voice technologies. The inclination to assign personality traits to these technologies suggests that machines may be processed along similar social and vocal dimensions as human voices. Given the increasing prevalence of voice technology in everyday tasks, this systematic review examines the factors in the psychology of voice acoustics that influence listeners’ trustworthiness perception of speakers, be they human or machine. Overall, this systematic review has revealed that voice acoustics impact perceptions of trustworthiness in both humans and machines. Specifically, combining multiple acoustic features through multivariate methods enhances interpretability and yields more balanced findings compared to univariate approaches. Focusing solely on isolated features like pitch often yields inconclusive results when viewed collectively across studies without considering other factors. Crucially, situational, or contextual factors should be utilised for enhanced interpretation as they tend to offer more balanced findings across studies. Moreover, this review has highlighted the significance of cross-examining speaker-listener demographic diversity, such as ethnicity and age groups; yet, the scarcity of such efforts accentuates the need for increased attention in this area. Lastly, future work should involve listeners’ own trust predispositions and personality traits with ratings of trustworthiness perceptions.

## 1 Introduction

Digitisation is changing the way modern societies interact and communicate. The use of artificial intelligence and speech synthesis has entered many domains of our daily life, such as autonomous vehicles, automated customer support, telehealth and companion robots, and smart home assistants. Considering that trust is a key factor in the acceptance of technology (Bryant et al., 2020; Large et al., 2019; Seaborn et al., 2022) as well as the healthy functioning of a flourishing society, it makes the multi-disciplinary research area of trustworthy voice acoustics of growing importance and relevance. Overall, existing literature suggests that speech acoustics influence first impressions of speakers’ perceived trustworthiness (Tsantani et al., 2016; Oleszkiewicz et al., 2017; Stewart and Ryan, 1982; Nass and Lee, 2000). Nonetheless, when biological, demographic, cultural, and situational factors are not adequately considered, the overall findings often remain inconclusive. To the best of our knowledge, this is the first systematic review that aims to understand the relationship between voice acoustics and attributions of trustworthiness in humans and machines.

### 1.1 The physiology of voice perception and speech acoustics

By merely hearing a stranger’s voice, such as a telemarketer, we tend to form instant impressions of their identity, discerning cues like gender, age, accent, emotional state, personality traits (e.g., perceived trustworthiness), and even hints about their health condition (cf. Nass and Brave, 2005; Kreiman and Sidtis, 2011). Voice, the carrier of speech, allows us to perceive human traits through auditory signals generated during speech production. Physiologically, during speech production, airflow from the lungs is transformed into sound waves by vocal fold vibrations in the larynx, and these waves are shaped by the vocal tract’s articulators, producing the diverse sounds of speech, cf. source-filter theory (Lieberman et al., 1992; Kamiloğlu and Sauter, 2021).

Table 1 exhibits certain acoustic features and how speech acoustics shape first impressions during social interactions (Bachorowski and Owren, 1995; Weinstein et al., 2018; Maltezou-Papastylianou et al., 2022; Shen et al., 2020; Cascio Rizzo and Berger, 2023). Voice quality features such as Harmonic-to-noise ratio (HNR), jitter, shimmer, cepstral peak prominence (CPP) and long-term average spectrum (LTAS) tend to be indicative of the perceived roughness, breathiness or hoarseness of a voice, often seen in vocal aging and pathologies research (Da Silva et al., 2011; Linville, 2002; Jalali-najafabadi et al., 2021; Farrús et al., 2007; Chan and Liberman, 2021). Moreover, past studies seem to suggest that each attributed speaker trait may follow a different time course in terms of stimulus duration (McAleer et al., 2014; Mahrholz et al., 2018; Lavan, 2023). For instance, dominance attributions seem to develop as early as 25 milliseconds (ms), while trustworthiness and attractiveness attributions are strengthened gradually over exposure periods ranging from 25 ms to 800 ms (Lavan, 2023).

**Table 1:** Summary characteristics of speech acoustics.

Acoustic features	Typically measured in…	Key characteristics
Fundamental frequency (F0); perceive as pitch.	Hertz (Hz)	F0 is the lowest rate of vocal fold vibrations, and F0 variability is usually captured by vocal intonation within an utterance. “Size or frequency code” theory (Ohala, 1983, 1995): Men’s lower pitch due to longer, thicker folds; women’s higher pitch due to shorter folds (Latinus and Belin, 2011; Frühholz and Schweinberger, 2021; Lavan et al., 2019). Average speaking frequencies: Men, 100–120 Hz; Women, 200–240 Hz; Children, 300 Hz (Mahendru, 2014; Schweinberger et al., 2014; Gelfand, 2017).
Amplitude; perceived as loudness.	Decibels (dB)	Indicative of air pressure variations.
Speech rate	Syllables per second (syll/s)	Typically estimated at about 4–6 syllables per second in English (Reetz and Jongman, 2020). “Effort code” theory (Gussenhoven, 2002): Faster speech rates shown to increase speakers’ perceived competence, credibility, trustworthiness and willingness to help (Yokoyama and Daibo, 2012; Smith and Shaffer, 1995; Rodero et al., 2014).
HNR	dB	Lower HNR signifies more noise in a voice signal (Fernandes et al., 2018; Ferrand, 2002). Noise in terms of voice, encompasses any component of the signal that interferes with the clarity, purity and overall quality of the intended speech signal. Typically, this noise is not harmonically related to the fundamental frequency of the voice, such as alterations in vocal fold tissue, muscle tension, respiratory patterns, or even ambient sounds and electronic interference (Ferrand, 2002). Older adults typically show slower speech rates, lower HNR, and differences in pitch and voice quality compared to younger adults (Lavan et al., 2019; Rojas et al., 2020; Heffernan, 2004; Ferrand, 2002; Baus et al., 2019; McAleer et al., 2014).
Jitter	%	Reveals micro-fluctuations in pitch caused by irregular vocal fold vibrations (Schweinberger et al., 2014; Baus et al., 2019; Felippe et al., 2006).
Shimmer	dB	Measures micro-fluctuations in amplitude, reflecting variations in voice intensity (Schweinberger et al., 2014; Baus et al., 2019; Felippe et al., 2006).
CPP	dB	A lower CPP is indicative of a breathy voice (Da Silva et al., 2011; Löfqvist, 1986; Hammarberg et al., 1980; Linville, 2002).
LTAS	dB	A lower LTAS often indicates longer vocal tract sizes (Da Silva et al., 2011; Löfqvist, 1986; Hammarberg et al., 1980; Linville, 2002), which are linked to deeper, more resonant voices associated with dominance, particularly observed in males (Gussenhoven, 2002; Puts et al., 2007).
Alpha-ratio	dB	Provides information about the distribution of energy across different frequency ranges (i.e., the ratio between low-frequency and high-frequency energy within a voice signal) (Sundberg et al., 2011; McAleer et al., 2014). It is often related to voice quality measures, such as the perceptual attributes of vocal effort, breathiness and vocal timbre (Chan and Liberman, 2021).
Mel-frequency cepstral coefficients (MFCCs)	Unitless	MFCCs are not voice signals themselves but derived from a multi-step process, including Fourier transformation, that provides a compact representation of the spectral properties of the voice signal (Zheng et al., 2001). They capture important information about the speech sounds while reducing the amount of data. MFCCs are widely used in various applications such as speech recognition systems, speaker identification, and emotion detection. They are also used in machine learning models to distinguish between high-quality and low-quality voice recordings, or to detect specific voice disorders when combined with other acoustic features (Rehman et al., 2024; Deng et al., 2024).

### 1.2 Definitions of trust and perceived trustworthiness

Trust has been shown to influence perceptions of first impressions (Freitag and Bauer, 2016), personal relationships (Ter Kuile et al., 2017), work performance (Brion et al., 2015; Lau et al., 2014), cooperation and sense of safety within communities (Castelfranchi and Falcone, 2010; Krueger, 2021). While extensive literature discusses trust models, most are theoretical (Harrison McKnight and Chervany, 2001; Mayer et al., 1995), offering varying definitions encompassing expected actions (Gambetta, 2000), task delegation (Mayer et al., 1995), cooperativeness (Yamagishi and Yamagishi, 1994; Yamagishi, 2003; Deutsch, 1960), reciprocity (Ostrom and Walker, 2003), and “encapsulated interest” (Maloy, 2009; Hardin, 2002; Baier, 2014). Current research tends to explore trust as either a single-scale or multi-dimensional concept, often focusing on the three-part relation of “A trusts B to do X,” within specific contexts (cf. Bauer and Freitag, 2018). Intrinsically, trustee B’s perceived trustworthiness to do X is shaped by trustor A’s dispositional, learned and situational trust factors, risk assessment and beliefs towards the trustee, such as gender stereotyping in relation to different occupations and contexts (Tschannen-Moran and Hoy, 2000; Smith, 2010; Seligman, 2000; Freitag and Bauer, 2016; Castelfranchi and Falcone, 2010). Furthermore, social trust formation tends to lean towards a dichotomised view, namely generalised and particularised trust (cf. Freitag and Traunmüller, 2009; Schilke et al., 2021; Uslaner, 2002). Overall, trusting someone or perceiving them as trustworthy can be expressed as the trustor’s reliance on a trustee (e.g., an individual, a community, an organisation or institution), with the belief or expectation of behaving in a manner that contributes to the trustor’s welfare (e.g., by assisting in the completion of a task) or at least not against it (e.g., sharing a secret). In turn, this helps support or induce a sense of mutual benefit between them, all the while, taking into account the situational context and the trustor’s predispositions.

Throughout this review, the terms trustor / listener / participant, and trustee / speaker may be used interchangeably.

### 1.3 Measuring trust propensity and perceived trustworthiness

Although there are a series of multi-disciplinary variations in past research aimed to capture the true essence of trust, it all boils down to two methods: (a) explicit measures of trust attitudes and behaviours through self-assessments using rating scales. These scales can be dichotomous (e.g., yes/no answers), probabilistic (i.e., ratings from 0 to 100%) or following a Likert scale format (Rotter, 1967; Knack and Keefer, 1997; Soroka et al., 2003); (b) implicit behavioural measures through the use of the prisoner’s dilemma game and the trust game experiment (also known as the investment game) derived from behavioural economics and games theory (Berg et al., 1995; Deutsch, 1960). Explicit measures of trust have also become a standardised practise in assessing one’s propensity to trust and perceived trustworthiness (Glaeser et al., 2000; Bauer and Freitag, 2018; Naef and Schupp, 2009; Kim, 2018).

Previous behavioural and cognitive research, including studies on voice perception and production, has emphasized the significance of sample sizes and research environments. Samples of 24–36 participants per condition tend to reliably yield high agreement between participant ratings (Lavan, 2023; McAleer et al., 2014; Mileva et al., 2020), while both online and lab-based experiments have provided comparable data quality (Del Popolo Cristaldi et al., 2022; Germine et al., 2012; Uittenhove et al., 2023; Honing and Reips, 2008).

### 1.4 Voice technology and the rise of intelligent agents

Humans naturally attribute social traits to others, including animals and even artificially intelligent entities (i.e., agents) like humanoid robots, virtual assistants, and chatbots. Consequently, research on human-agent interaction (HAI) emphasizes studying human behaviour for designing interactive intelligent agents (IAs), with voice playing a crucial role in attributing social traits, as seen in the “Computers as Social Actors” (CASA) paradigm (Nass et al., 1994; Lee and Nass, 2010; Seaborn et al., 2022). The “uncanny valley” phenomenon further illustrates this, describing the uneasiness felt when an IA looks or sounds almost human but not quite (Mori, 1970; Mori et al., 2012).

Speech production in technological settings tends to refer to either canned speech (i.e., unchangeable pre-recorded speech samples) or synthesised speech, both seen in voice research (Nass and Brave, 2005; Kaur and Singh, 2023; Clark et al., 2019; Cambre and Kulkarni, 2019; Weinschenk and Barker, 2000; Kang and Heide, 1992). Past studies in HAI have revealed a positive relationship between perceptions of trustworthiness, rapport, learning and vocal entrainment (i.e., adapting one’s vocal features to sound more similar to the person they are talking to) (Cambre and Kulkarni, 2019). Further studies supporting the effects of voice acoustics in IAs and trustworthiness have observed (1) a connection between vocal pitch and trustworthiness (Elkins and Derrick, 2013), (2) a preference towards more “natural” humanlike IA voices (Seaborn et al., 2022), and (3) the influence of the similarity-attraction effect. The similarity-attraction effect exhibits a preference and more positive attitudes towards speakers that are perceived to be more similar to the participant (Nass and Brave, 2005; Dahlbäck et al., 2007; Nass and Lee, 2000; Clark et al., 2019). For instance, Dahlbäck et al. (2007) observed a preference towards voice-based IAs that matched the listeners’ own accent regardless of the IA’s actual level of expertise, strengthening the case of people assigning human traits and predispositions to IAs.

Therefore, trustworthiness perceptions in voice-based IAs mirror those in human voices. Accordingly, trustors’ dispositional, learned, and situational trust towards IAs, alongside IAs’ perceived competence and ease of use should also be taken into account. Additional factors affecting trustworthiness attributions like perceived risk, especially regarding security, privacy, and transparency, also hold significance (Razin and Feigh, 2023), often examined through models such as the Technology Acceptance Model (TAM) and its variations (cf. Riener et al., 2022; Nam and Lyons, 2020).

Finally, trust propensity in HAI is often measured using scales like the Negative Attitudes to Robots (NARS) (Nam and Lyons, 2020; Jessup et al., 2019). Overall, measurements of trustworthiness perceptions in HAI tend to follow the same methods laid out in the previous section with some alterations to match the technological aspect. For instance, sometimes a Wizard of Oz experiment is conducted for implicit measures, where during HAI the researcher either partly or fully operates the agent, while the participant is unaware, thinking the agent acts autonomously (Dahlbäck et al., 1993; Riek, 2012).

### 1.5 Motivation

Given the above, this systematic review attempts to consolidate the existing multi-disciplinary literature on voice trustworthiness in both human and synthesised voices. Specifically, this review aims to address the question of “how do acoustic features affect the perceived trustworthiness of a speaker?,” while also reviewing participant demographics, voice stimuli characteristics and task(s) involved.

## 2 Methods and analysis

This systematic review followed the Preferred Reporting Items for Systematic Reviews and Meta-Analyses (PRISMA) checklist (Page et al., 2021a,b). The search was performed on the 31^st^ of October 2022, and all studies were initially identified by electronic search. Searches were repeated on the 18^th^ January 2024 to identify any additional publications. A pre-registration protocol has been created for this review on the Open Science Framework (OSF^1^) under the CC-by Attribution 4.0 International license.

This review adopted a narrative synthesis approach, to consolidate findings across studies investigating vocal trustworthiness in human speakers and voice-based IAs. The decision to use narrative synthesis was informed by the research objective, which focused on identifying and summarising acoustic features, demographic characteristics, and task paradigms across studies, rather than deriving effect sizes or pooled estimates. This approach allowed for a comprehensive examination and categorisation of findings into themes to identify trends, gaps, and contextual nuances in the literature, and inform future research directions.

### 2.1 Search strategy

Five bibliographic databases (Scopus, PsycInfo, ACM, ProQuest, PubMed) were searched using tailored search syntax detailed in Table 2, guided by the question: “How do acoustic features affect the perceived trustworthiness of a speaker?.” Queries, developed collaboratively by all authors, have focused on English-language records published until January 18, 2024, using Boolean operators and wildcards for optimal search. Additional records were identified through manual searches, citation chaining, and exploration of Scholar database, books, and conference proceedings.

**Table 2:** Search query syntax used in bibliographic databases.

Database	Search query syntax
Scopus	(TITLE-ABS-KEY (trust*) AND TITLE-ABS-KEY (voice OR vocal* OR prosod* OR speech OR acoustic* OR utter* OR speaker$ OR praat OR pitch OR “fundamental frequency” OR hnr OR “harmonic$-to-noise” OR “voice quality” OR accent*) AND TITLE-ABS-KEY (adult$))
PsycInfo	AB trust* AND AB (voice OR vocal* OR prosod* OR speech OR acoustic* OR utter* OR speaker OR praat OR pitch OR “fundamental frequency” OR HNR OR “harmonics-to-noise” OR “voice quality” OR accent*) AND AB adult
ACM	[Abstract: trust*] AND [[Abstract: voice] OR [Abstract: vocal*] OR [Abstract: prosod*] OR [Abstract: speech] OR [Abstract: acoustic*] OR [Abstract: utter*] OR [Abstract: speaker?] OR [Abstract: praat] OR [Abstract: pitch] OR [Abstract: “fundamental frequency”] OR [Abstract: hnr] OR [Abstract: “harmonic?-to-noise”] OR [Abstract: “voice quality”] OR [Abstract: accent*]]
ProQuest	summary(trust*) AND summary(voice OR vocal* OR prosod* OR speech OR acoustic* OR utter* OR speaker$ OR praat OR pitch OR “fundamental frequency” OR HNR OR “harmonic$-to-noise” OR “voice quality” OR accent*) AND summary(adult$)
PubMed	(trust*[Title/Abstract]) AND (voice[Title/Abstract] OR vocal*[Title/Abstract] OR prosod*[Title/Abstract] OR speech[Title/Abstract] OR acoustic*[Title/Abstract] OR utter*[Title/Abstract] OR speaker[Title/Abstract] OR praat[Title/Abstract] OR pitch[Title/Abstract] OR “fundamental frequency”[Title/Abstract] OR HNR[Title/Abstract] OR “harmonics-to-noise”[Title/Abstract] OR “voice quality”[Title/Abstract] OR accent* [Title/Abstract]) AND (adult[Title/Abstract])

### 2.2 Eligibility criteria for screening and selection of studies

Full-text papers have been obtained for titles and abstracts deemed relevant, based on specified inclusion and exclusion criteria. Papers were independently screened by CMP and SP, and any discrepancies were resolved through discussion.

Studies were included if: (a) participants were adults, irrespective of ethnicity, nationality, age and gender; (b) the study design involved a quantitative or mixed-methods approach; and (c) examined variables and reported outcomes focused on the acoustic characteristics of a speaker, with respect to their perceived trustworthiness.

Studies were excluded if: (a) reported outcomes did not focus on acoustic cues in relation to perceptions of trustworthiness of a human or IA; (b) characteristics of participants, stimuli and tasks involved could not be obtained; (c) the study design followed a qualitative-only approach; and (d) only the abstract was written in English, while the main paper was written in a language other than English.

### 2.3 Data extraction

Extracted information was divided into three categories accompanied by the publication’s title and a reference key: (a) study characteristics, containing data such as the author, publication year, country that the study has taken place, number of participants, the aim of the study, vocal cues examined, task(s) involved, analyses and outcome; (b) listener characteristics, relating to the demographics of participants; (c) stimuli characteristics, including details of the stimulus itself and speaker demographics.

### 2.4 Risk-of-bias assessment method

The methodological quality and risk of bias of the included studies were assessed using a tailored scoring rubric adapted from Leung et al. (2018). The assessment evaluated risk of bias across five domains: conceptual clarity, reliability, internal validity, external validity, and reproducibility. Each domain covers specific criteria, scored from 0 to 2 points (0 = high risk of bias, 1 = moderate risk of bias, 2 = low risk of bias), detailed in Supplementary material. The maximum possible score for a study was 18 points (9 criteria × 2 points). The findings from the risk of bias assessment can be found in the Results section. Note that, such risk-of-bias scales do not necessarily reflect the quality of the evidence collected and used in the respective studies per se, or the reliability or quality of the studies involved more generally. Rather, they reflect “risk” in terms of how and what appears presented in the final publications, as filtered through the present authors’ ability to extract these points from the respective manuscripts in the structured manner dictated by the scoring tool.

## 3 Results

### 3.1 Quantity of research available

**Figure 1:**
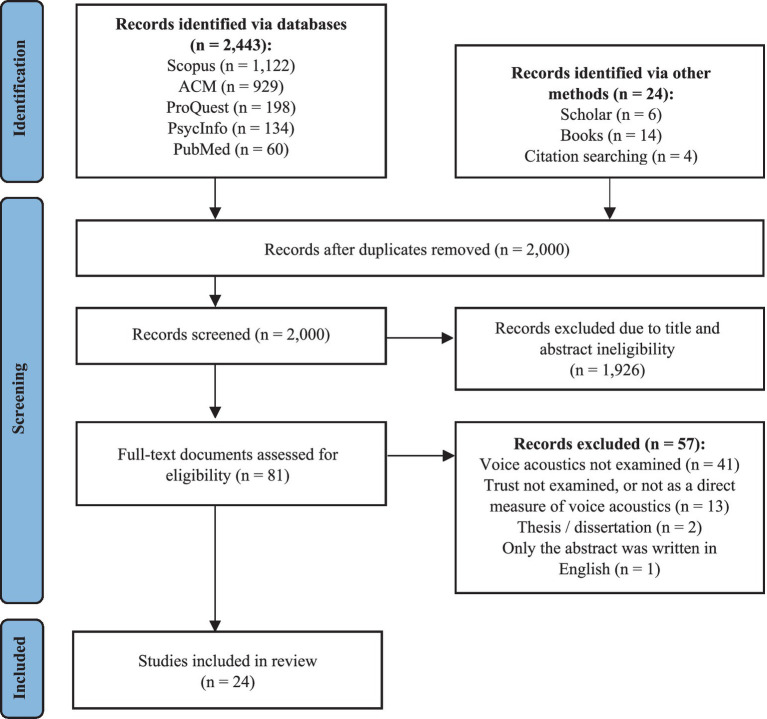
Identification of included studies in the systematic review, following the PRISMA flow diagram (Page et al., 2021a,b).

Electronic and hand searches have identified 2,467 citations, of which 2,000 unique ones have been screened via Rayyan software (Ouzzani et al., 2016). Following elimination of duplicates, 81 potentially relevant citations remained. After full-text review and application of inclusion criteria, 57 citations have been excluded, resulting in 24 eligible studies (see Figure 1).

The 24 studies have been published between 2012 and 2024 and were conducted across Europe, America and Asia—nine in the UK, six in the US, two in Poland and one study each in France, Canada, China, Japan and Singapore, while two remain unclear (see Table 3). Eight of those are conference proceedings (Torre et al., 2016; Tolmeijer et al., 2021; Muralidharan et al., 2014; Maxim et al., 2023; Lim et al., 2022; Kim et al., 2023; Elkins et al., 2012; Klofstad et al., 2012) and the remaining 16 are journal publications. Among them, 14 studies have focused on perceived trustworthiness in terms of human speakers and the remaining 10 in terms of voice-based IAs. Twenty-one studies have focused on the effects of vocal pitch or pitch-related features with 12 of them incorporating the additional properties of pitch range, intonation, glide, formant dispersion, harmonic differences, HNR, jitter, shimmer, MFCCs, alpha ratio, loudness, pause duration and speech rate (see Table 4). Four studies solely focused on either speech duration or speaking rate.

**Table 3:** Descriptive statistics of the total sample size averaged between all included studies.

	Mean	Median	SD	Mode	Min	Max
Human speaker studies						
Listeners	346.3	181	625	85, 40	40	2,538
Speakers	42	25	51	64	1	208
Voice-based IA studies						
Listener	108.2	86	69.3	None	30	234
Speakers	3	2	3.5	1	1	12

**Table 4:** Summary of all included studies.

Study	Country	Study design			Vocal cues examined and outcome (i.e., more trustworthy when…)									Risk-of-bias score^1^
		Task	Theme	Analyses	Duration	Pitch	Intonation pattern	Amp	HNR	Jitter	Shimmer	Speech rate	Additional notes	
Studies: perceived trustworthiness of human speakers.														
Groyecka-Bernard et al. (2022)	Poland	Explicit 7-point	Generic	Regression	+	N/A	N/A	N/A	N/A	N/A	N/A	N/A	Gender-irrelevant outcome.	14
Schirmer et al. (2020)	Singapore	Explicit 7-point	Public comms	Regression	N/A	-	N/A	+	-	+	+	+	Younger and female speakers. Amplitude = intensity range.	16
Mileva et al. (2020)	UK	Explicit 9-point	Public comms	Correlation	N/A	NSR	N/A	NSR	NSR	NSR	NSR	NSR	NSR for formant dispersion too.	12
Baus et al. (2019)		Explicit 9-point	Generic	PCA Regression	N/A	NSR	NSR	N/A	+	NSR	NSR	N/A	NSR for formant dispersion, glide and alpha ratio too. + HNR for Scottish speakers only.	14
Belin et al. (2019)	UK	Explicit	Generic	t-test	N/A	NSR	+	N/A	N/A	N/A	N/A	N/A	+ for intonation pattern of higher pitch at the start and end of an utterance and lower in the middle.	13
Ponsot et al. (2018)	France	Explicit	Generic	Regression ANOVA	N/A	+	+	N/A	N/A	N/A	N/A	N/A	Gender-irrelevant outcome.	8
Mahrholz et al. (2018)	UK	Explicit	Generic	Correlation Regression	+	N/A	N/A	N/A	N/A	N/A	N/A	N/A	Stronger gender correlations (M > F).	15
O'Connor and Barclay (2018)	Canada	Explicit 2AFC & 7-point	Social behav.	ANOVA	N/A	- (P) + (A)	N/A	N/A	N/A	N/A	N/A	N/A	Male speakers only. P = prosocial A = antisocial	11
Mileva et al. (2018)	UK	Explicit 9-point	Generic	ANOVA	N/A	NSR	N/A	N/A	N/A	N/A	N/A	N/A		14
Oleszkiewicz et al. (2017)	Poland	Explicit 7-point	Generic	Regression	N/A	-	N/A	N/A	N/A	N/A	N/A	N/A	Gender-irrelevant outcome.	12
Tsantani et al. (2016)	UK	Explicit 2AFC	Generic	T-test ANOVA	N/A	-	N/A	N/A	N/A	N/A	N/A	N/A	Gender-irrelevant outcome.	11
McAleer et al. (2014)	UK	Explicit 9-point	Generic	PCA Regression	N/A	+ (M)	+ (F)	N/A	- both sexes	NSR	NSR	N/A	M / F = gender. - glide for females. NSR for formant dispersion & alpha ratio too.	13
Klofstad et al. (2012)	US	Explicit 2AFC	Public comms	t-test	N/A	-	N/A	N/A	N/A	N/A	N/A	N/A	Gender-irrelevant outcome.	14
Yokoyama and Daibo (2012)	Japan	Explicit	Public comms	ANCOVA	N/A	N/A	N/A	N/A	N/A	N/A	N/A	+		8
Studies: Perceived trustworthiness of voice-based IA.														
Deng et al. (2024)	China	Mixed 7-point	Safety proced.	ANOVA Regression	NSR	-	-	NSR	N/A	N/A	N/A	NSR	Listener vocal response measured. NSR for formants. + MFCC.	13
Maxim et al. (2023)	US	Explicit 7-point	Tele-health	ANOVA	N/A	NSR	N/A	-	N/A	N/A	N/A	-	Trend towards lower pitch.	12
Kim et al. (2023)	US	Mixed 7-point	Safety proced.	Mancova	N/A	+	+	N/A	N/A	N/A	N/A	+		8
Goodman and Mayhorn (2023)	US	Mixed 7-point	Tele-health	Correlation t-test	N/A	NSR	N/A	N/A	N/A	N/A	N/A	N/A	Female IA preference.	16
Lim et al. (2022)	UK	Explicit 7-point	Cust. service	Binomial Correlation Qualitative	N/A	+	N/A	+	N/A	N/A	N/A	+	Trust-propensity was measured too.	12
Tolmeijer et al. (2021)	US	Mixed 5-point	Cust. service	Non-parametric t-test and ANOVA	N/A	NSR	N/A	N/A	N/A	N/A	N/A	N/A		8
Torre et al. (2020)	UK	Mixed 7-point	Finance services	Regression	N/A	+	N/A	N/A	N/A	N/A	N/A	N/A		12
Torre et al. (2016)	UK	Implicit	Finance services	Regression	N/A	+	N/A	N/A	N/A	N/A	N/A	+	Speech rate = articulation rate.	12
Muralidharan et al. (2014)		Mixed	Cust. service	ANOVA	N/A	+	N/A	N/A	N/A	N/A	N/A	N/A	Significance for lower time delay (flanging). Pitch = pitch range.	9
Elkins et al. (2012)	US	Implicit	Safety proced.	Regression	N/A	+	N/A	N/A	N/A	N/A	N/A	N/A	Listener vocal response measured. + response time. Less prominent effects over time.	15

NSR, no statistical-significance reported; +/−, higher/lower.

1 Higher scores denote lower “risk” (out of a maximum possible score of 18 points)—see the relevant Methods section for an explanation of what this measures.

Green cells highlight statistically significant correlations and associated directionality (+/−) between acoustic features and perceived trustworthiness, whereas red cells highlight non-significant or inconclusive results (NSR).

Most studies used Likert scales, typically in the rage of 1–7, to assess perceived trustworthiness (see Table 4). Some employed implicit decision tasks, while others combined explicit and implicit measures. Regression models, including linear mixed models and logistic regression, were common for exploring vocal acoustics and trustworthiness. Pearson’s correlations assessed relationship strength. ANOVA, t-tests, and occasionally PCA or mixed methods were used for analysis.

Only one study examined age-group differences, i.e., adults older and younger than 60 years old (Schirmer et al., 2020). As seen in Tables 3, 5, 11 studies had fewer than 100 participants (Schirmer et al., 2020; Elkins et al., 2012; Mileva et al., 2020; Ponsot et al., 2018; Mileva et al., 2018; Oleszkiewicz et al., 2017; O'Connor and Barclay, 2018; Deng et al., 2024; Goodman and Mayhorn, 2023; Muralidharan et al., 2014), six with up to 50 (Goodman and Mayhorn, 2023; Mileva et al., 2018; Oleszkiewicz et al., 2017; Muralidharan et al., 2014; Kim et al., 2023; Ponsot et al., 2018). Ten studies had 100–550 participants (Lim et al., 2022; Tolmeijer et al., 2021; Torre et al., 2020; Baus et al., 2019; Mahrholz et al., 2018; McAleer et al., 2014; Yokoyama and Daibo, 2012; Belin et al., 2019; Maxim et al., 2023; Klofstad et al., 2012), while one had over 2,000 (Groyecka-Bernard et al., 2022). Most used audio-only stimuli, but seven used audio-visual (Yokoyama and Daibo, 2012; Elkins et al., 2012; Lim et al., 2022; Mileva et al., 2020; Maxim et al., 2023; Deng et al., 2024; Mileva et al., 2018). Five studies created over 100 usable stimuli (Groyecka-Bernard et al., 2022; Mahrholz et al., 2018; Schirmer et al., 2020; Ponsot et al., 2018; Torre et al., 2016) (see Table 6).

**Table 5:** Participant characteristics of all included studies.

Study	Adjusted sample size	Gender	Mean age [range]	Additional participant details
Studies: Perceived trustworthiness of human speakers.				
Groyecka-Bernard et al. (2022)	2,538	46% males; 54% females.	32.51	N/A
Schirmer et al. (2020)	80	25% younger males; 25% younger females; 25% older males; 25% older females.	23.7 [20–32 years] (younger males); 21.1 [19–27 years] (younger females); 67.9 [60–91 years] (older males); 68 [60–77 years] (older females).	Older adults: 2 with normal hearing (<= 25 dB); 28 with slight hearing impairment (26–40 dB); 9 with moderate impairment (41–60 dB); 1 with severe impairment (61–80 dB) that was corrected with a hearing aid.
Mileva et al. (2020)	99	7% males; 93% females.	19 [18–50 years]	N/A
Baus et al. (2019)	279 (study 1); 258 (study 2).	33% males (S1); 67% females (S1); 50% males (S2); 50% females (S2).	20.2 (S1); 22.03 (S2).	Spanish nationality.
Belin et al. (2019)	500	29% males; 71% females.	Median age = 24 [19–65 years]	N/A
Ponsot et al. (2018)	44 (study 1; trust task = 23); 40 (study 2; trust task = 19).	52% males (S1 trust task); 48% females (S1 trust task); 47% males (S2 trust task); 53% females (S2 trust task).	22 (S1 trust task); 21 (S2 trust task).	N/A
Mahrholz et al. (2018)	181	24% males; 76% females.	21.3 [18–27 years] (males); 20.1 [18–30 years] (females).	Scottish nationality.
O'Connor and Barclay (2018)	85 (study 1); 63 (study 2).	100% females (S1 & S2)	18.21 (S1); 18.9 (S2).	N/A
Mileva et al. (2018)	40	20% males; 80% females.	20.1 [18–30 years]	N/A
Oleszkiewicz et al. (2017)	50	20% blind males; 34% blind females; 16% sighted males; 30% sighted females.	37.9 [24–64 years] (healthy blind adults); 38.7 [24–65 years] (sighted adults).	N/A
Tsantani et al. (2016)	40 (study 1); 240 (study 2).	33% males (S1); 67% females (S1); 24% males (S2); 76% females (S2).	24 (S1); 20 (S2).	N/A
McAleer et al. (2014)	320	37% males; 63% females.	28.5	N/A
Klofstad et al. (2012)	210	50% males; 50% females.	Undergraduate students	N/A
Yokoyama and Daibo (2012)	466	53% males; 47% females.	19.6	N/A
Studies: Perceived trustworthiness of voice-based IA.				
Deng et al. (2024)	75	23% males (group 1 & 2); 25% females (group 1); 29% females (group 2).	22.69 [19–27 years] (group 1); 22.15 [19–26 years] (group 2).	N/A
Maxim et al. (2023)	165	56% males; 43% females; 1% non-binary.	43.35 [24–68 years]	144 white; 9 Asian; 5 black; 7 mixed-race.
Kim et al. (2023)	30	50% males; 50% females.	21 [18–38 years]	N/A
Goodman and Mayhorn (2023)	47	55% males; 38% females; 2% non-binary; 5% undisclosed.	19.5	N/A
Lim et al. (2022)	202	60% males; 38% females; 2% non-binary.	28.11 [18–60 years]	N/A
Tolmeijer et al. (2021)	234	41% males.	33 [19–74 years]	US nationality.
Torre et al. (2020)	108	22% males; 78% females.	19 [18–48 years]	British nationality.
Torre et al. (2016)	83	38% males; 62% females.	Median age = 21 [18–67 years]	British nationality = 5 from Wales and the rest from across England.
Muralidharan et al. (2014)	50 (study 1); 23 (study 2).	39% males (S2); 61% females (S2).	[18–28 years]	N/A
Elkins et al. (2012)	88	60% males; 40% females.	25.45	N/A

The “adjusted sample size” column notes the total number of participants after having excluded any individuals from the analyses.

**Table 6:** Stimuli characteristics of all included studies.

Study	Stimuli	Speaker demographics
Studies: Perceived trustworthiness of human speakers.		
Groyecka-Bernard et al. (2022)	1,248 audio-only stimuli; 60 Polish-language WAV files per listener; Sampling rate = 96 kHz; Resolution = 16-bit.	208 Polish speakers; 52% males, 48% females; Mean age = 32.83.
Schirmer et al. (2020)	520 audio-only stimuli; 2 sentences × 13 expressions × 20 speakers.	20 Singaporean native English speakers with acting experience; Younger adults: 25% males, 25% females; Mean age = 23.8 (males), 22.2 (females); Older adults: 25% males, 25% females; Mean age = 63 (males), 69.2 (females).
Mileva et al. (2020)	22 audio-visual stimuli; 7 stimuli from females; Mean duration = 3.41 s.	22 speakers; 32% females.
Baus et al. (2019)	Audio-only stimuli; Study 1: 64 Spanish recordings of the word “Hola”; mean duration: males = 319 ms; females = 338 ms; normalised; Study 2: 64 recordings, re-used from McAleer et al. (2014).	Study 1: 64 Spanish; 50% males; Mean age = 22.1; Study 2: 64 Scottish voices, re-used from McAleer et al. (2014).
Belin et al. (2019)	Audio-only stimuli; Re-synthesised and manipulated pre-existing Scottish voice stimuli of the word “hello” from McAleer et al. (2014); Split between low and high trustworthiness as per the rating results obtained by McAleer et al. (2014).	Subset of Scottish male and female voices, re-used from McAleer et al. (2014).
Ponsot et al. (2018)	Audio-only stimuli; Study 1: ~700 trials × 2 genders of the French word “bonjour”; Study 2: 420 stimuli (20 French words, including “bonjour” × 7 pitch contour filters × 3 repetitions); For all stimuli: sampling rate = 44.1 kHz; Resolution = 16-bit mono; Normalisation range = 75–80 dB.	Study 1: 2 French speakers; 1 male (aged 28); 1 female (aged 29); Study 2: 12 French speakers; 50% males, 50% females; Mean age = 33.33 [21–57 years].
Mahrholz et al. (2018)	120 audio-only stimuli; Lab-based WAV recordings; 2 durations (word/sentence) × 2 contexts (with/without context); Sampling rate = 44.1 kHz; Resolution = 16-bit mono; Normalised; Average duration: males = 411.1–3,019.6 ms; females = 394.6–3,172.8 ms.	60 Scottish; 50% males, 50% females; Mean age = 23.2 (males), 20.2 (females).
O'Connor and Barclay (2018)	Audio-only stimuli; Paired words × 2 contexts (prosocial/antisocial) × 2 genders (feminised = higher pitch; masculinised = lower pitch).	4 speakers; 100% males; Mean age = 18.
Mileva et al. (2018)	40 audio-visual stimuli; 2 genders × 2 pitch conditions (higher/lower); males: Higher-pitch = 140 Hz, lower-pitch = 90 Hz; females: higher-pitch = 250 Hz, lower-pitch = 170 Hz.	20 speakers; 50% males; Mean age = 23.
Oleszkiewicz et al. (2017)	Audio-only stimuli; WAV format with higher/lower pitch manipulation; Sampling rate = 96 kHz; Resolution = 32-bit; Normalisation = 70 dB.	8 speakers; 50% males, 50% females.
Tsantani et al. (2016)	66 audio-only stimuli per study, Re-used from McAleer et al. (2014); 2 pitch conditions (higher/lower, 20 Hz shift) × 2 contexts/studies (backward/forward speech manipulation); Average duration = 400 ms.	33 Scottish voices, re-used from McAleer et al. (2014); 55% males, 45% females.
McAleer et al. (2014)	64 audio-only stimuli; WAV format with neutral tone of voice of the word “Hello”; Sampling rate = 44.1 kHz; Resolution = 16-bit mono; Average duration = 319 ms (males), 390 ms (females).	64 Scottish; 50% males; Mean age = 28.2.
Klofstad et al. (2012)	54 audio-only stimuli; 2 genders × 2 pitch conditions (higher / lower); Sampling rate = 44.1 kHz; Amplitude normalised; Mean pitch = 187 Hz females, 107 Hz males.	27 speakers; 37% males, 63% females; Mean age = 33 [20–55 years] (males), 31 [21–60 years] (females).
Yokoyama and Daibo (2012)	4 audio-visual stimuli; 2 gaze states (high = 8% looking at the camera; low = 83%) × 2 speech rates (faster = 510 syllables per minute; slower = 330).	1 Japanese, female speaker; 23 years old.
Studies: Perceived trustworthiness of voice-based IA.		
Deng et al. (2024)	Audio-visual stimuli. Participant responses were recorded and stored for speech analysis in relation to perceived trustworthiness in HAI.	Automated-vehicle system with audio-visual interaction features and voice recognition features.
Maxim et al. (2023)	2 audio-visual stimuli; 1 agent × 1 scenario × 2 voice characteristics (1 extroverted and 1 introverted); Extroverted agent: Speech rate = 216 words per minute; Base pitch = 140 Hz; Introverted agent: Speech rate = 184 words per minute; Base pitch = 84 Hz; Volume = 15% less (−1.41 dB) than the extroverted voice.	A female embodied conversational agent.
Kim et al. (2023)	2 audio-only stimuli; “Urgent” vs. “calm” voice; Urgent voice = faster speech rate, higher pitch, variable intonation; Calm voice = slow speech rate, static intonation.	Recorded human voices.
Goodman and Mayhorn (2023)	6 audio-only stimuli; 3 × pitch conditions (high/intermediate/low).	2 synthesised voices; 1 male, 1 female.
Lim et al. (2022)	2 audio-visual stimuli; 2 × personalities (extroversion = higher pitch, speech rate, volume; introversion = lower pitch, speech rate, volume).	An embodied conversational agent.
Tolmeijer et al. (2021)	5 audio-only stimuli; 2 genders × 2 pitch conditions (higher/lower); 1 gender ambiguous voice = pitch shifted towards the average of high-pitch female and low-pitch male voices.	A voice assistant using a US accent; 1 male, 1 female and 1 gender-ambiguous voice.
Torre et al. (2020)	40 audio-only stimuli; 2 intents (neutral/amused); Sentence length = 16.6 syllables.	4 British females in their 20s; Birmingham accent = 50% speakers; SSBE accent = 50% speakers.
Torre et al. (2016)	240 audio-only stimuli; 4 blocks of 20 sentences per speaker; Mean number of syllables per sentence = 16.95.	12 British females in their 20s; Plymouth accent = 25% speakers; Birmingham accent = 25% speakers; London accent = 25% speakers; SSBE accent = 25% speakers.
Muralidharan et al. (2014)	Audio-only stimuli; 5x pitch range conditions = 525 Hz (humanlike), 395 Hz, 195 Hz, 125 Hz, 1 Hz (machine-like).	2 synthesised voices; 1 male, 1 female.
Elkins et al. (2012)	Audio-visual stimuli; 4 questions × 2 genders × 2 demeanors (neutral / smiling). Participant responses were recorded and stored for F0 analysis, resulting to a total of 866 WAV files with a final sampling rate of 11.025 kHz.	1 embodied conversational agent, portraying both male and female audio-visual aspects independently.

As indicated in the “Theme” column of Table 4, all 24 studies have been assigned a thematic (i.e., contextual) category based on shared situational attributes to provide more clarity and relevance during the discussion of their findings. Specifically, during the review stage, the situational factors of each study were examined. These factors were derived from either the study’s inherent task (e.g., customer-barista interaction or fire warden simulation scenarios) or the meaning conveyed by the uttered stimuli (e.g., election speech, or generic greeting). They played a key role in qualitatively grouping studies that shared similar situational contexts. For instance, the “public communication” theme has examined interactions involving public speaking in conferences (Yokoyama and Daibo, 2012), student elections (Mileva et al., 2020), or a political context (Schirmer et al., 2020; Klofstad et al., 2012). This iterative process was aimed to uncover consistent patterns and variations in how vocal acoustic features like pitch, amplitude, and intonation influence trustworthiness perceptions within specific, similar situational contexts.

Ultimately, seven distinct thematic categories were derived from this approach. These categories spanned a spectrum from generic first impressions, such as greetings and factual statements (Baus et al., 2019; Belin et al., 2019; McAleer et al., 2014; Mileva et al., 2018; Ponsot et al., 2018; Tsantani et al., 2016; Groyecka-Bernard et al., 2022; Mahrholz et al., 2018; Oleszkiewicz et al., 2017), to specific domains such as public communication (Schirmer et al., 2020; Klofstad et al., 2012; Yokoyama and Daibo, 2012; Mileva et al., 2020), social behaviour (O'Connor and Barclay, 2018), customer service (Tolmeijer et al., 2021; Muralidharan et al., 2014; Lim et al., 2022), financial services (Torre et al., 2020; Torre et al., 2016), telehealth advice (Goodman and Mayhorn, 2023; Maxim et al., 2023) and safety procedures (Kim et al., 2023; Deng et al., 2024; Elkins et al., 2012).

### 3.2 Risk-of-bias assessment findings

The total risk of bias scores for the 24 reviewed studies ranged from 8 to 16 out of a maximum of 18 points, with a mean, median and mode of 12 (SD = 2.5). Eight studies (33%) scored between 14 and 16 points, 12 studies (50%) scored between 9 and 13 points, and four studies (17%) scored 8 points (see Table 4).

Conceptual clarity was a consistent domain of weakness, with only six studies providing a clear and explicit definition of trust or trustworthiness (Deng et al., 2024; Elkins et al., 2012; Goodman and Mayhorn, 2023; Kim et al., 2023; Lim et al., 2022; Muralidharan et al., 2014). The majority relied on implicit or vague conceptualisations, potentially limiting the interpretability and comparability of findings across studies. Reliability demonstrated notable variation, with only nine studies (38%) achieving the maximum score of 4 for using validated tools for measuring acoustic features and reporting intra- or inter-rater reliability (Baus et al., 2019; Goodman and Mayhorn, 2023; Elkins et al., 2012; Klofstad et al., 2012; Mahrholz et al., 2018; Schirmer et al., 2020; McAleer et al., 2014; Mileva et al., 2018; Mileva et al., 2020).

Majority of studies scored highly on internal validity due to clear randomisation or pseudo-randomisation procedures, stimuli quality and justified sample sizes. External validity emerged as a widespread limitation, with only three studies (13%) scoring highly for diverse speaker and listener samples (Baus et al., 2019; Schirmer et al., 2020; Oleszkiewicz et al., 2017). Most studies were restricted to narrow demographic groups. Reproducibility was a strength, with 19 studies (75%) earning maximum scores due to detailed methodological descriptions.

Overall, the assessment highlighted strengths in the reproducibility domain and weaknesses in the domains of conceptual clarity and external validity. Greater attention to defining trust and trustworthiness, diversifying speakers and listeners, and improving methodological transparency is needed to strengthen the robustness and applicability of future research. For more information, see Tables 4–6, while the full scoring criteria and explanations for individual study scores are available in Supplementary material.

## 4 Discussion

In this review, vocal pitch has emerged as a predominant focus across all 24 included studies, followed by investigations into amplitude, intonation, HNR, jitter, shimmer, speech duration, and/or speech rate. To facilitate a comprehensive discussion, findings have been categorised into sections on human speakers and voice-based IAs, grouping relevant studies accordingly.

The interpretation of study outcomes has been significantly shaped by contextual factors, leading to the qualitative grouping of studies into thematic (i.e., contextual) categories. Each thematic category summarises findings on acoustic features and their implications for perceptions of trustworthiness within specific contexts or situations, as detailed further in the discussion. For instance, studies within the “telehealth advice” theme have examined trustworthy voice acoustics in scenarios involving medication guidance and mental wellness practices. This thematic approach has facilitated the identification of consistent patterns and variations in how vocal acoustic features contribute to communication dynamics and shape perceptions of trustworthiness within specific contexts. Without these situational considerations, the overall findings across studies seemed to be inconclusive.

In total, seven contextual themes have been identified (also see Table 4). Three of these themes are evident in human speaker studies: “generic first impressions” (e.g., from greetings to factual statements), “public communication,” and “social behaviour.” The remaining four themes are identified in voice-based IA studies: “customer service,” “financial services,” “telehealth advice,” and “safety procedures.” For a summary of findings see Table 7.

**Table 7:** Summary of trust-related acoustic features in human and IA studies: Actionable insights for practitioners and recommendations for future research.

Theme	Trustworthy acoustic features	Limitations	Recommendations and insights
Studies: Perceived trustworthiness of human speakers.			
Generic first impressions	*Pitch*: In English contexts, a higher pitch or rising intonation often seems to boost trustworthiness perceptions, albeit mixed findings in non-English settings. *Voice quality*: Native English listeners often favour lower HNR, while non-native listeners may prefer higher HNR for vocal clarity or precise enunciation. *Speech duration*: Longer segments (~2–3 s) allow more processing time, enhancing trustworthiness.	Primarily English-speaking samples; limited cross-cultural research. Some multimodal designs (face + voice) complicate pure acoustic findings. Conflicting pitch results can arise from different task types (Likert vs. forced-choice).	*For researchers*: Compare short vs. long utterances in diverse languages and speaker demographics. *For practitioners (e.g., marketers, voice coaches)*: In English contexts, use slightly longer greetings plus moderate/higher pitch for a friendly first impression, checking cultural fit in non-English contexts.
Public communication	*Pitch*: A lower pitch can convey authority or dominance in both male and female speakers, depending on cultural norms. *Voice quality*: Younger or more expressive voices (e.g., increased jitter/shimmer) can be favoured, but cultural preferences vary. *Speech rate*: A faster rate suggests competence/expertise (“effort code” theory).	Highly varied contexts (political speeches, conferences, elections) limit universal generalisation, since each environment has its own norms, audience expectations, and stakes. Biases based on demographic diversity (e.g., age, ethnicity, gender) remain under-explored (e.g., preference for younger/female voices). Some studies combine vocal with facial cues.	*For researchers*: Conduct single-modality (voice-only) tests to isolate acoustic influences, and then compare with multimodal tasks (audio-visual). Investigate for different speaker-listener demographics, cultures and languages. *For practitioners (e.g., speakers and trainers)*: Use a moderately faster rate to project competence and a slightly lower pitch for authority—mindful of local and cultural norms, and audience preferences (e.g., age, gender).
Social behaviour	*Pitch*: In pro-social contexts, lower-pitched male voices are deemed more trustworthy; in antisocial contexts, a higher pitch can reduce perceived aggression or intimidation. Aligns with “frequency code” theory: lower pitch = dominance, higher pitch = submission/non-threat.	Only one study specifically contrasting pro- vs. antisocial male voices. Cultural, age and gender nuances beyond male speakers remain under-explored. Other acoustic features (loudness, speech rate, voice quality) rarely examined here.	*For researchers*: Replicate with broader demographics (e.g., female, non-Western speakers-listeners) and varied social contexts. Examine pitch synergy with other acoustic and voice quality features. *For practitioners (e.g., campaign strategists)*: In altruistic messaging, lower-pitched male voices may be deemed as trustworthiness. However, in negative or conflict scenarios, a slightly higher pitch may soften intimidation.
Studies: Perceived trustworthiness of voice-based IAs.			
Customer service	*Pitch*: Mixed or inconclusive; some data suggest higher pitch helps, others find no effect. *Speech rate & loudness*: Faster, louder voices often project competence and extroversion. *Time delay (flanging)*: A delay beyond ~0.01 s yields a “machine-like” sound, reducing trust. *Synergy*: Higher pitch + faster rate + louder volume can signal enthusiasm, while lower pitch + longer delay appears unnatural.	Different methods (audio vs. audio-visual) produce varied pitch outcomes. Small samples or limited speaker diversity reduce generalisability. Gender stereotyping manipulations not always generalisable.	*For researchers*: Investigate how different acoustic cues interact globally (e.g., Western vs. Asian markets) to capture global variations and IA personas. Conduct A/B tests to see how minor pitch/rate tweaks affect warmth, competence, and trust. *For practitioners (e.g., chatbot / voice-tech scientists and product managers)*: Use a moderately faster speech rate and louder tone for high-stakes support scenarios (e.g., billing disputes or quick issue resolutions) to convey urgency and competence. For a personalised, friendly brand, adopt moderately higher pitch and faster speech for an enthusiastic tone—or personalise based on users’ mood and personality. Avoid lower pitch with steady cadence, as it risks sounding mechanical or impersonal. Limit flanging (i.e., avoid speech delays >0.01 s) and robotic intonations to ensure the voice sounds human and engaging. Track user metrics (satisfaction, conversation duration, etc.). If distrust arises, tweak acoustics gradually and retest.
Financial services	*Pitch & speech rate*: Higher pitch + faster articulation in female-sounding IAs often associated with perceived happiness, helpfulness, competence (humans ascribe personality traits to the voice).	Mainly female British accents; potential cultural and demographic biases. Predominantly investment tasks; unsure if findings extend to other financial contexts (insurance, loans, etc.).	*For researchers*: Examine if pitch and speech rate preferences hold for male voices too. Assess if a higher pitch and a faster speech rate is effective beyond investment contexts (e.g., insurance, banking, etc.). *For practitioners (e.g., robot-advisor scientists and developers)*: For virtual advisors, consider using a slightly higher pitch with faster articulation for competence and positive traits—be aware of accent preferences. Track conversation outcomes through real-time analytics (e.g., abandonment rates, user satisfaction). If trust declines, tweak pitch or speed gradually, then retest with A/B experiments.
Telehealth advice	*Pitch, speech rate, loudness*: A lower pitch, slower rate, and softer volume often convey empathy, especially in female voices. *Listener traits*: Extroverted listeners may trust IAs more regardless of acoustic settings, indicating individual differences may override vocal features.	Typically, small samples and varied methodologies; some purely audio, others multimodal.	*For researchers*: Develop consistent trust metrics for telehealth IAs. Investigate user personality traits (e.g., extroversion vs. introversion). For practitioners (e.g., mental health app and companion robot designers): For a remote triage and guidance service, providers could adopt a gentler profile (lower pitch, slower rate, softer volume) to foster a caring, professional vibe—mindful of individual differences. Similarly, for personal therapy session, consider adaptive voice settings (e.g., pitch level, speech rate) that can be fine-tuned to patient demographics or preferences (e.g., older adults, mental health patients).
Safety procedures	*Synergy*: Higher pitch + faster rate + varied intonation + varied MFCC + higher intensity in listeners’ responses often linked to boosted immediate trust in emergencies (fire alarms, driving instructions). Associated to feeling more invested in HAI. Trust may fade over time as urgency subsides or listeners gain more information.	Limited speakers/scenarios (often short stimuli). Long-term trust or repeated exposure seldom explored. IAs’ acoustic features not examined.	*For researchers*: Examine if an “urgent voice” remains effective over prolonged or repeated alerts. Include speaker diversity (age, gender, ethnicity) for broader applicability. For practitioners (e.g., emergency system designers): For immediate hazard warnings (e.g., earthquake, road hazards), adopt higher pitch with a faster speech rate to convey urgency—then reduce intensity once people start following instructions. Alternatively, offer tiered voice prompts, where the first alert is highly urgent, followed by calmer updates to sustain trust without alarm fatigue.

### 4.1 The role of acoustic cues in the perceived trustworthiness of human speakers

Thirteen of the 24 studies have focused on perceived trustworthiness of adult human voices. Six have solely assessed pitch-related measures (Mileva et al., 2018; Tsantani et al., 2016; O'Connor and Barclay, 2018; Oleszkiewicz et al., 2017; Belin et al., 2019; Ponsot et al., 2018), four have combined pitch with HNR, jitter, shimmer, loudness, formant dispersion, or speech rate (Baus et al., 2019; McAleer et al., 2014; Schirmer et al., 2020; Mileva et al., 2020), two have focused solely on speech duration (Groyecka-Bernard et al., 2022; Mahrholz et al., 2018), and one on speaking rate (Yokoyama and Daibo, 2012).

All studies have used explicit measures like rating scales, with 7-point (Groyecka-Bernard et al., 2022; Schirmer et al., 2020; O'Connor and Barclay, 2018; Oleszkiewicz et al., 2017) and 9-point (Baus et al., 2019; McAleer et al., 2014; Mileva et al., 2018; Mileva et al., 2020) Likert scales being common. Analyses have included correlational, inferential, and regression models (details in Table 4). While some studies have linked trustworthiness to lower or higher pitch independent of gender, others have noted gender’s influence. Building on the premise of situational factors, the following part of this subsection presents a discussion on study findings, categorised thematically according to contextual similarities.

#### 4.1.1 “Generic first impressions” theme

Nine of the studies on human voice trustworthiness have focused on generic first impression scenarios, using a variety of audio stimuli (e.g., greetings such as the word “hello,” or snippets from The Rainbow Passage (Fairbanks, 1960)). The main aspects that have been studied under this theme include pitch and related features like intonation and glide (Baus et al., 2019; McAleer et al., 2014; Oleszkiewicz et al., 2017), and some have also considered voice quality features (Baus et al., 2019; Belin et al., 2019; McAleer et al., 2014; Mileva et al., 2018; Ponsot et al., 2018; Tsantani et al., 2016). Two studies specifically, have only analysed speech duration (e.g., comparison between shorter and longer sentences or words) (Groyecka-Bernard et al., 2022; Mahrholz et al., 2018).

##### 4.1.1.1 Vocal pitch and related features

Current findings have primarily suggested a positive link between pitch, rising intonation at both ends of a stimulus and trustworthiness attributions in English-speaking contexts (McAleer et al., 2014; Belin et al., 2019). Nevertheless, cultural differences seem to be prevalent, as mixed findings for pitch have been identified for non-English speaking studies (Baus et al., 2019; Ponsot et al., 2018; Oleszkiewicz et al., 2017). Multimodal research (i.e., faces and voices) has also yielded inconclusive results regarding pitch’s impact, noting that there may be a stronger influence of faces in such cases (Mileva et al., 2018). Moreover, methodological differences seem to have played a role in the current findings: English-speaking studies using Likert scales have favoured higher pitch for trustworthiness, whereas research utilising a 2AFC task (Tsantani et al., 2016) has deemed lower pitch as more trustworthy. Further research comparing these methodologies is necessary for a clearer understanding.

##### 4.1.1.2 Voice quality features

Significant findings have centered on HNR, revealing cultural disparities based on English-speaking stimuli: native listeners seem to favour lower HNR for trustworthiness (McAleer et al., 2014), whereas non-native listeners seem to prefer higher HNR (Baus et al., 2019), regardless of the speaker’s gender. Voice quality features tend to be sensitive in respect to voice quality pathologies and physiological changes that occur in aging (Farrús et al., 2007; Felippe et al., 2006; Ferrand, 2002; Rojas et al., 2020; Jalali-najafabadi et al., 2021), which may account for these preferences. For instance, native listeners may gravitate more towards youthful-sounding voices, which may promote more positive or upbeat impressions. In contrast, non-native listeners, may prioritise vocal clarity and precision in foreign speech that usually comes with a higher HNR. Considering that cross-cultural vocal trustworthiness studies seem to be scarce, further investigations are warranted for a more comprehensive understanding.

##### 4.1.1.3 Temporal features

Both studies examining speech duration have indicated that longer stimuli, around 2–3 s, tend to be perceived as more trustworthy than shorter ones, e.g., a vowel or a word (Groyecka-Bernard et al., 2022; Mahrholz et al., 2018). However, one of them (Mahrholz et al., 2018) has added that even stimuli as short as 0.5 s can convey trustworthiness, consistent with previous research (Lavan, 2023; McAleer et al., 2014). Moreover, these perceptions appear to be consistent across cultures, such as Polish (Groyecka-Bernard et al., 2022) and Scottish (Mahrholz et al., 2018) speakers. A potential explanation for these findings may relate to longer speech duration potentially allowing for more thorough processing, thus influencing trust perceptions, as well as introducing more opportunities for response variability among listeners (Groyecka-Bernard et al., 2022). Having said that, further cross-cultural studies are still needed for definitive conclusions.

#### 4.1.2 “Public communication” theme

Four studies seem to fall under this theme category, which either tackle trustworthiness judgments in terms of public speaking in conferences (Yokoyama and Daibo, 2012) and student elections (Mileva et al., 2020), or in terms of stimuli with a political context (Schirmer et al., 2020; Klofstad et al., 2012).

##### 4.1.2.1 Temporal features

One of those studies (Yokoyama and Daibo, 2012) has assessed trustworthiness perceptions based solely on the speech rate of a female speaker in Japan, finding a preference for faster speech. Despite using Singaporean English speakers and listeners, a second study has reached similar conclusions (Schirmer et al., 2020). In support of these findings, past research, including the “effort code” theory, suggest that faster speech rates tend to convey greater knowledge and expertise (Smith and Shaffer, 1995; Rodero et al., 2014; Gussenhoven, 2002). Consequently, boosting speakers’ perceived confidence, credibility, and persuasiveness, particularly in public speaking contexts. Additionally, these findings may also be indicative of listeners’ preference towards younger speakers, considering that slower speech rate tends to be more associated with aging (Schirmer et al., 2020).

##### 4.1.2.2 Voice quality features

The aforementioned Singaporean study (Schirmer et al., 2020) has also shown a preference for voices with lower pitch and HNR, but higher jitter, shimmer, and intensity range. This is the only study that has explicitly explored age differences, revealing a preference for younger speakers and a general preference for female speakers across ages. The contradictory lower HNR, higher jitter and shimmer preferences though, may stem from perceived expressiveness or individual and cultural influences on vocal aesthetic preferences. Conversely, a UK study under this theme (Mileva et al., 2020) has yielded inconclusive results, potentially due to their multimodal design (faces and voices). Their multimodality makes it more difficult for a direct comparison with the previous, unimodal (i.e., voice-only) studies, and to interpret their findings.

##### 4.1.2.3 Vocal pitch and related features

Lastly, two studies (Schirmer et al., 2020; Klofstad et al., 2012) have exhibited a preference for lower-pitched voices regardless of gender, which may potentially be influenced by individual and cultural norms of vocal aesthetic appeal. An alternative interpretation for lower-pitched female voices may be that they sound more dominant and thus, perceived as more authoritative, confident, and competent (Ohala, 1983; Klofstad et al., 2012).

#### 4.1.3 “Social behaviour” theme

##### 4.1.3.1 Vocal pitch and related features

The only study under this theme has explored male voices in pro-social and anti-social scenarios (O'Connor and Barclay, 2018). Lower-pitched voices have been noted as more trustworthy in positive contexts and higher-pitched voices in negative contexts. These observations were partly explained in terms of higher pitch potentially mitigating the perceived intimidation of antisocial behaviour in men (O'Connor and Barclay, 2018). This seems to align with the “frequency code” theory, where higher-pitched voices tend to signal smaller body sizes, primarily seen in women and children; thus potentially conveying a friendlier or less threatening demeanour (Ohala, 1983; Ohala, 1995).

Altogether, vocal cues in human voices seem to play a significant role in trustworthiness attributions, albeit influenced by contextual factors. It is further suggested that vocal cues may have stronger effects when voice acts as the sole or primary modality for drawing trustworthiness inferences.

### 4.2 The role of acoustic cues in the perceived trustworthiness of voice-based IAs

The remaining 11 studies in this review focused on assessing the perceived trustworthiness of voice-based Intelligent Agents (IAs), whether using synthesised or pre-recorded human voices. Similar to human speakers, voice-based IAs are often evaluated with human behaviour in mind, with context also playing a significant role. Contextual themes and associated acoustic features for trustworthy speech are discussed further.

#### 4.2.1 “Customer service” theme

Three voice-based IA studies examining trustworthiness attributions fall under this theme category. Contexts vary from barista scenarios (Lim et al., 2022) to task-assistance scenarios (Tolmeijer et al., 2021; Muralidharan et al., 2014).

##### 4.2.1.1 Vocal pitch and related features

Findings on pitch have been inconclusive, which may partly stem from differences in study designs; one study used audio-visual stimuli with correlational analyses (Lim et al., 2022), while the other two employed audio-only stimuli with inferential models (Tolmeijer et al., 2021; Muralidharan et al., 2014). Tolmeijer et al. (2021) has also focused extensively on gender-stereotyping, manipulating synthetic voices to sound more masculine, feminine, or gender-ambiguous. The lack of pitch significance in trustworthiness perceptions in these studies, suggests that listeners may not rely solely on pitch for voice-based IAs in assistive roles. These findings challenge the importance of vocal pitch in shaping trustworthiness perceptions of IAs.

##### 4.2.1.2 Vocal pitch in combination with other acoustic features

Past research (Muralidharan et al., 2014) has suggested that combining pitch and flanging (i.e., speech time delay manipulation) influences trustworthiness perceptions. They have found that a lower pitch range with greater time delay tends to be perceived as more machine-like and less trustworthy compared to natural human speech. They added that human speech typically has a natural time delay of about 0.01 s, and increasing this delay can make it sound less natural. This deviation, along with a less animated voice, may lead to uneasiness in listeners, supporting theories on social inferences from HAI (Mori, 1970; Mori et al., 2012; Nass et al., 1994; Muralidharan et al., 2014).

Furthermore, a louder voice with a faster speech rate and higher pitch tends to be perceived as more trustworthy, supporting theories linking trust formation with positive traits (Lim et al., 2022). Faster speech rate tends to portray speakers’ deeper understanding and passion for the subject. In combination with higher pitch it is usually associated with extroversion and openness (Ohala, 1995; Lim et al., 2022; Maxim et al., 2023), further portraying speakers as competent, persuasive, and credible (Yokoyama and Daibo, 2012; Smith and Shaffer, 1995; Rodero et al., 2014; Gussenhoven, 2002). Only one study has examined listeners’ trust propensity, revealing positive and negative associations with trustworthiness attributions dependent on the scales used (Lim et al., 2022). Overall findings under this theme seem to be appropriate if we interpret them as listeners being more accepting and trusting of speakers’ assistance on a task. Nonetheless, more extensive research is needed in this area before these findings can be deemed as generalisable.

#### 4.2.2 “Financial services” theme

Both studies (Torre et al., 2020; Torre et al., 2016) in this theme employed implicit investment tasks, with one also using a 7-point Likert scale (Torre et al., 2020). Both have assessed female-only voices with various British accents and used regression models for analysis.

##### 4.2.2.1 Vocal pitch in combination with other acoustic features

Findings have indicated that higher pitch and faster articulation rate seem to be associated with more trustworthiness. Additionally, they have linked higher pitch to positive emotions such as happiness. These findings seem to align with past research linking greater articulatory effort to higher perceptions of knowledge, confidence, and helpfulness (Gussenhoven, 2002). The preference for higher-pitched voices in female IAs strengthens the case of attributing human traits to IAs, as women typically have higher-pitched voices due to physiological factors. Past research has also exhibited a preference for higher-pitched women, linking them with positive traits like attractiveness and trustworthiness (Lavan, 2023; McAleer et al., 2014). The current findings may also strengthen the case for humans assigning gender roles to assistive occupations, even in HAI (Tolmeijer et al., 2021).

#### 4.2.3 “Telehealth advice” theme

Two studies have explored trustworthiness judgments in receiving advice for medication (Goodman and Mayhorn, 2023) and mental wellness (Maxim et al., 2023) contexts.

##### 4.2.3.1 Vocal pitch in combination with other acoustic features

While one has focused on vocal pitch of male and female IA using audio-only, the other has examined pitch, speech rate, and loudness of a female IA with audio-visual stimulus. Despite no reported acoustic significance for trustworthiness, a trend towards lower pitch, speech rate, and volume in female voices is observed. Additionally, extroverted listeners have offered higher ratings overall, irrespective of speakers’ perceived traits (Maxim et al., 2023).

Authors seem to have attributed these observations to voice similarity with mental health professionals, suggesting softer, empathetic, and confident perceptions (Maxim et al., 2023). Moreover, slower speech rate and lower volume, which are often associated with physiological changes occurring in aging (Lavan et al., 2019; Rojas et al., 2020; Heffernan, 2004; Ferrand, 2002; Baus et al., 2019; McAleer et al., 2014). As such, speakers may have also been perceived as older and probably more knowledgeable. These findings further highlight HAI drawing inferences from human-human interactions and linking trustworthiness to positive traits. Nonetheless, limited stimuli and differing methodologies between the two studies may affect their generalizability. For instance, Maxim et al. (2023) examined the similarity-attraction effect among other aspects and employed a multi-modal design (i.e., faces and voices), which makes it more difficult for a direct comparison with the second, unimodal (i.e., voice-only) study (Goodman and Mayhorn, 2023), and to interpret their findings.

#### 4.2.4 “Safety procedures” theme

The last three studies on voice-based IAs explored attributions of trustworthiness employing scenarios such as security screening (Elkins et al., 2012), fire warden simulation (Kim et al., 2023) and voice assistance during driving simulation (Deng et al., 2024).

##### 4.2.4.1 Vocal pitch in combination with other acoustic features

All three studies have associated higher vocal pitch with increased trustworthiness in voice-based IAs, albeit varying in their methodology. Two of them have assessed trustworthiness through participants’ verbal responses during HAI (Elkins et al., 2012; Deng et al., 2024). They have reported that higher-pitched responses with greater pitch and MFCC variability, higher intensity, and longer response time may correspond to higher trustworthiness ratings. These findings may relate to participants developing more positive perceptions of the IA, in terms of dominance, authoritativeness and competence, and feeling more invested during HAIs as per the “effort code” theory (Ohala, 1983; Klofstad et al., 2012; Gussenhoven, 2002). However, these effects seem to diminish with prolonged HAI, possibly due to the accumulation of information and the opportunity to make further inferences over time (Elkins et al., 2012). While these studies provide valuable insights, pre-assessing participants’ trust propensity and personality traits could enhance conclusions. The final study (Kim et al., 2023), which examined the acoustics of voice-based IAs instead, has similarly reported that higher pitch with faster speech rate and variable intonation has prompted higher trustworthiness ratings, labelling that combination of acoustics as an “urgent voice.”

Granted that these three studies have offered limited stimuli, which like previously mentioned, might not be sufficient to draw generalised conclusions to the broader population. Nevertheless, despite methodological variances, all of them have consistently reported similar results. This consistency may be attributed to the heightened vocal urgency observed in speakers during emergency situations, which could also be perceived as more authoritative, eager to assist, and concerned with everyone’s safety (Yokoyama and Daibo, 2012; Smith and Shaffer, 1995; Rodero et al., 2014; Gussenhoven, 2002).

All things considered, vocal cues of voice-based IAs seem to be playing a significant role in attributions of trustworthiness. However, contextual and situational factors are equally prevalent in this section as in research on human voices, enhancing the interpretability of findings. It is further highlighted the influence of human-human interactions and social inferences from human behaviour when studying HAIs. Finally, majority of the HAI studies had less than a hundred participants (Goodman and Mayhorn, 2023; Muralidharan et al., 2014; Elkins et al., 2012; Torre et al., 2016; Kim et al., 2023; Deng et al., 2024), and only one study had more than 5 speakers (Torre et al., 2016) making their findings potentially more difficult to generalise to the wider population, even though they were reported to be well-powered.

### 4.3 Limitations and the future of research on trustworthy voices

The 24 papers identified in this review, represent the body of existing research in relation to speech acoustics and perceptions of trustworthiness. Our conclusions are drawn from a comprehensive synthesis of all available evidence.

Studies varied in participant numbers, with 13 involving less than 100 participants and 6 of those having less than 50 (see Table 5). Regarding speakers, most studies had 5 or fewer speakers, with 8 having 60 or fewer; see Table 6 for a summary of the stimuli and Table 3 for the descriptive statistics of participants and speakers across all reviewed studies. While participant sample sizes may appear limited, past research supports sample sizes of 24–36 per condition (Lavan, 2023; McAleer et al., 2014; Mileva et al., 2020). Most studies have used explicit, self-reported tasks, with some attempting real-life scenario recreation for additional behavioural data. More effort may be needed for capturing a wider range of contexts.

Most studies have relied on convenience sampling from student populations, raising concerns about demographic diversity and external validity. This sampling approach may not represent the broader population, potentially impacting the generalisability of findings. Consequently, variations in sample size and recruitment methods could have contributed to the polarised research outcomes identified, with a potential bias towards younger white generations. Moreover, online experiments have been proposed as viable alternatives to lab-based studies, offering comparable data quality and potentially better generalisability and ecological validity depending on the research question and recruitment characteristics (Del Popolo Cristaldi et al., 2022; Germine et al., 2012; Uittenhove et al., 2023; Honing and Reips, 2008).

Future research should address limitations in sample characteristics of both speakers and listeners to enhance demographic diversity and generalisability. Methodological limitations of existing studies should be acknowledged and addressed to improve the reliability of reported outcomes. Additionally, future research should explore the relationship between perceived trustworthiness based on listeners’ voice ratings and their trust propensity, as well as individual differences in listeners and speakers. Cross-examinations should be expanded to include a wider range of demographic factors such as age, accents, ethnicity, and nationality, while also considering their disposition towards trust. Rigorous mixed-methods study designs should be employed to provide comprehensive insights into the effects of past and current behaviours on trustworthiness perceptions from voice acoustics, ensuring conclusive findings. Moreover, current research lacks studies examining speakers’ own self-perceptions of producing trustworthy speech, which could complement existing literature on listeners’ trustworthiness attributions.

Furthermore, the qualitative thematic categorisation has highlighted disparities in the depth of exploration on voice trustworthiness across different situational contexts. While themes like generic first impressions (Baus et al., 2019; Belin et al., 2019; McAleer et al., 2014; Mileva et al., 2018; Ponsot et al., 2018; Tsantani et al., 2016; Groyecka-Bernard et al., 2022; Mahrholz et al., 2018; Oleszkiewicz et al., 2017) seem to have received substantial attention, others such as telehealth advice (Goodman and Mayhorn, 2023; Maxim et al., 2023), financial services (Torre et al., 2020; Torre et al., 2016) and customer service (Tolmeijer et al., 2021; Muralidharan et al., 2014; Lim et al., 2022) seem to be comparatively under-explored. This highlights the need for future research to address these gaps and expand our understanding of how vocal acoustic features influence trustworthiness perceptions across diverse contexts.

Overall, this systematic review highlights both shared and unique aspects of how trustworthiness is perceived in human voices and voice-based IAs. For human voices, judgements of trustworthiness emerge from a complex blend of acoustic features, social inferences, and interactional context. In contrast, voice-based IAs rely more on engineered acoustic profiles, yet they, too, are often evaluated along human-like social dimensions. As shown in Tables 4, 7, factors such as pitch, speech rate, loudness, and voice quality can be tuned to elicit or reduce trust, with different combinations proving more effective in specific scenarios (e.g., faster, louder delivery for customer service; slower, softer voices for telehealth). Moreover, Table 7 consolidates common acoustic features across both human and IA voices, demonstrating how certain cues, when appropriately balanced, can transcend medium or modality to influence trustworthiness perceptions.

Given these overlapping mechanisms, the need for comparative research on human and IA voices is more pressing than ever. Trust remains central to social cohesion and collaboration; thus, as voice-based IAs increasingly permeate telehealth (e.g., mental health triaging, companion robots or wellbeing apps), customer service (e.g., call centre chatbots, dispute resolution voice-based IAs), financial services (e.g., AI-driven robot advisors, voice-based personal budgeting IAs, automated insurance underwriting), and even self-driving vehicles (e.g., real-time hazard alerts and route guidance), there is a growing need to adapt these technologies so they inspire and sustain user trust—see Table 7 for actionable insights per industry. Moreover, since everyday tasks now blur the boundaries between human and machine interactions, understanding how we attribute trust to non-human voices is both academically significant and practically essential. A dual focus on human and synthesised voices can offer valuable insights into the cognitive processes guiding trust judgements, ultimately shaping the development of more effective, natural-sounding AI voices. By aligning voice design more closely with human-like trust cues, these systems will be better equipped to function ethically and efficiently in an increasingly technological society.

## 5 Conclusion

This paper has systematically reviewed 24 studies to explore the impact of vocal acoustics on perceived trustworthiness in both human speakers and voice-based IAs, shedding light on human behaviour and attitudes toward vocal communication.

In summary, acoustic features appear to correlate with trustworthiness judgments in both human and IA voices, albeit they may exert more pronounced effects when the voice serves as the sole or predominant modality for inferring trustworthiness. Moreover, their effects are best understood within their intended contexts for enhanced interpretability. Overall, pitch seems to be influential when assessed in combination with other acoustic features, while as a sole factor it appears to be less reliable. Additionally, HAI seems to draw social inferences from human-human interactions, listeners’ trust propensity and personality traits. Hence, highlighting the importance of studying these factors side by side.

To conclude, a comprehensive approach is needed to advance research on voice trustworthiness for more robust and well-rounded insights, as discussed in more detail in the limitations section of the discussion. Firstly, by considering dispositional and situational trust attitudes alongside current measures. Secondly, by cross-examining individual differences and demographic diversity in speaker-listener samples. Thirdly, there seems to be a gap in existing research regarding studies that explore speakers’ self-perceptions of delivering speech with trustworthy intent, a facet that could complement the existing literature on listeners’ attributions of trustworthiness. Lastly, by expanding the study of voice trustworthiness across diverse situational contexts, researchers can deepen insights into communication nuances and trustworthiness perceptions in contexts that have been less frequently investigated. See Table 7 for a more detailed summary of findings, paired with actionable insights for practitioners and recommendations for future research.

In closing, this review serves as a valuable reference for policymakers, researchers, and other interested parties. It offers insights into the current state of research while highlighting existing gaps and suggesting directions for future multi-disciplinary investigations.

## Data Availability

The original contributions presented in the study are included in the article/Supplementary material, further inquiries can be directed to the corresponding author.

## Footnotes

^1^ https://osf.io/cbps5/
